# Analysis of Pharmacist Interventions to Reduce Medication-Related Problems in a Neonatal Clinical Care Unit

**DOI:** 10.3390/pharmacy14020040

**Published:** 2026-03-02

**Authors:** Stephanie W. K. Teoh, Tamara Lebedevs, Geena Dickson, Marcus Femia, Nabeelah Mukadam

**Affiliations:** Pharmacy Department, King Edward Memorial Hospital, 374 Bagot Road, Subiaco, WA 6008, Australia; tamara.lebedevs@health.wa.gov.au (T.L.); geena.dickson@health.wa.gov.au (G.D.); marcus.femia@health.wa.gov.au (M.F.); nabeelah.mukadam@health.wa.gov.au (N.M.)

**Keywords:** pharmacy, medication safety, neonatology, medication related problem, pharmacist intervention

## Abstract

(1) Background: Medication-related problems (MRPs) are a significant burden on health care systems. Pharmacists play an important role in preventing and reducing MRPs through clinical review, education, and policy governance. This study analyzed pharmacist interventions within a 92-bed neonatal clinical care unit to better understand MRPs and guide targeted medication safety initiatives. (2) Methods: All pharmacist interventions documented in REDCap^®^ between 1 July 2022 and 30 June 2025 were analyzed identifying MRP incidence, types, and acceptability following interventions. (3) Results: A total of 873 pharmacist interventions were recorded during the study period. The most common MRPs were related to dosing errors (320/873, 36.7%), compliance with hospital policy (152/873, 17.4%), no indication apparent (106/873, 12.1%), drug interactions (66/873, 7.6%), and inadequate laboratory monitoring (40/873, 4.6%). Of these, 545/873, 62.4% were accepted by prescribers, while 228/873, 26.1% had unknown outcomes at the time of data entry. 343/873, 39.3% of interventions documented were from the Neonatal Intensive Care Unit, involving medications such as gentamicin (*n* = 46/343, 13.4%), benzylpenicillin (*n* = 37/343, 10.8%), caffeine (*n* = 34/343, 9.9%), parenteral nutrition (*n* = 23/343, 6.7%), and morphine (*n* = 16/343, 4.7%) and meropenem (*n* = 16/343, 4.7%)). (4) Conclusions: Regular analysis of pharmacist interventions provides valuable insights into local MRP trends and highlights opportunities for quality improvement and education.

## 1. Introduction

Medication-related problems (MRPs) are a significant burden on health care systems [1]. Pharmacists play an important role in preventing and reducing MRPs through clinical review, education, and policy governance [2]. Published literature indicates that pharmacist practice in the Neonatal Intensive Care Unit (NICU) involves diverse roles, including patient medication chart review, therapeutic drug monitoring, and the provision of medication information, which enables the prevention and monitoring of MRPs [3,4,5,6,7].

Systematic review and meta-analysis involving hospitals across different countries have shown that pharmacist clinical review was associated with reductions in the overall rate of medication errors occurrence, and improved patient outcomes [8,9,10]. The participation of pharmacists in ward rounds and review of patients’ prescriptions prior to dispensing was recommended in studies reviewing medication errors and strategies to improve patient safety in neonatal intensive care [7,10,11]. Multifaceted educational intervention was reported to have contributed to a significant reduction in the preparation and administration error rate [12,13,14,15].

Neonatal patients are vulnerable to medication errors due to the increased need for complex dose calculations, multiple medication dilutions and manipulations, and the frequent involvement of off-label use of medications [13,16]. The medication-use process within neonatal patients is more complex and has greater consequences resulting from error [17]. Errors due to patient misidentification and overdosing were particularly prevalent in neonates, with 47% of administration errors involving at least tenfold overdoses [17]. The use of medication protocols in the intensive care units is widely recommended to reduce medication errors and adverse drug events [18]. This is particularly useful for the NICU, where a high proportion of medicines are used off-label and where evidence-based medication information is sparse due to a limited number of clinical trials evaluating efficacy, dosage, and safety within the neonatal population [19,20].

Studies highlighted that pharmacist contribution to clinical review of total parenteral nutrition (TPN) regimens and patient medication chart review is beneficial to patient outcomes [3]. This is consistent with the previous study result demonstrated by the study site [21]. Pharmacist interventions should be regularly reviewed due to the complexity of medication use in neonates, the high frequency use of high-risk medications, and the potential for serious adverse events resulting from even minor medication errors [16]. Ongoing evaluation of these interventions is imperative in supporting healthcare systems and clinicians in understanding, implementing, and strengthening strategies aimed to reduce neonatal medication errors [22].

This is a single-center study aimed to evaluate the incidence and types of MRPs, and evaluate the acceptability and actions taken following pharmacist interventions at a tertiary NICU.

## 2. Materials and Methods

### 2.1. Study Site

The study hospital is a tertiary women’s and newborn hospital where more than 6000 births take place annually. It is the only major referral center in the state for high-risk pregnancies. The neonatal unit provides intensive, high dependency, and special care for preterm and sick newborn babies, with 2300 to 2400 neonatal admissions each year. The neonatal unit has 92 beds, including 30 intensive care beds (Clinical Significance Framework [CSF] level 6) and 62 special care beds (20 CSF level 5 and 42 CSF level 4) [23,24].

The clinical pharmacy service provided to the neonatal unit includes medication reconciliation, assessment of current medication management, clinical review of medication prescribed on the medication chart, therapeutic drug monitoring, TPN review, and facilitating continuity of medication management on discharge or transfer, as recommended by the Advanced Pharmacy Australia clinical pharmacy standards [2]. The number of medication charts reviewed by the clinical pharmacists is documented daily as part of the departmental key performance indicators.

The neonatal pharmacist also promotes medication safety through participation in weekly grand rounds, medication incident trend reporting, and delivery of multidisciplinary education. Additionally, the pharmacist coordinates the review and publication of neonatal medication protocols (*n* = 156), which are accessed by the study hospital and other neonatal units across the state [25].

### 2.2. Analysis of Pharmacist Interventions Documented

At the study hospital, pharmacists documented clinical interventions daily. From 2001 to 2015, interventions were documented manually using a paper-based system when performing clinical pharmacy services and were subsequently transcribed into an Excel^®^ spreadsheet and analyzed annually [21]. The pharmacist intervention documentation was transitioned to an advanced Microsoft Excel^®^ spreadsheet in 2016, with a Clinical Intervention (CI) documentation matrix guide (including the MRP (classifications) developed based on the Pharmaceutical Society of Australia (PSA) and the Society of Hospital Pharmacists of Australia (SHPA) model for CI documentation Matrix [26]. In 2022, an online platform on REDCap was introduced to improve efficiency in data entry and data analysis [27]. Since 1 July 2022, the pharmacists at the study hospital have been documenting pharmacy clinical interventions on a REDCap^®^ database, which is easily accessible by most electronic devices with secure data storage and access [27]. Information documented includes Staff Name, Intervention Details, Medication Details, Clinical Intervention Matrix [26], and Risk Analysis.

All neonatal pharmacist interventions documented in REDCap between 1 July 2022 and 30 June 2025 were analyzed, identifying MRP incidence, types, and acceptability following interventions. A risk analysis of the potential impact of interventions was completed using the Australian Standards for Risk Management. This risk analysis identified the severity of an MRP, which was determined by the potential consequence (impact) and likelihood of recurrence if the pharmacist’s intervention was not made. The use of risk analysis of pharmacist interventions was validated in previous studies [21,26].

The clinical pharmacy intervention data documented in REDCap^®^ was integrated with Power BI^®^ to generate an interactive dashboard, enabling streamlined and efficient reporting without delays or the need for manual data manipulation [27]. The dashboard was utilized as a tool to facilitate data analysis in this study.

## 3. Results

A total of 873 pharmacist interventions were recorded during the 3-year study period from 1 July 2022 to 30 June 2025. The 12-month intervention rate from 1 July 2024 to 30 June 2025 was 373 interventions per 12,032 medication charts reviewed, equivalent to 3.1 interventions per 100 medication charts reviewed.

### Frequency of Medication-Related Problems, Medications Involved, and Actions Taken Following Pharmacist Interventions

The most common MRPs were related to dosing errors (320/873, 36.7%), compliance with hospital policy (152/873, 17.4%), no indication apparent (106/873, 12.1%), drug interactions (66/873, 7.6%), and inadequate laboratory monitoring (40/873, 4.6%) (Table 1).

Following the identification of MRPs, the pharmacist recommendations were: Refer to prescriber (454/873, 52%), increase or decrease dose or amend dose schedule (189/873, 21.6%), and provision of information, including education, written summary of medications (85/873, 9.7%) (Table 2).

Of the recommendations made by the pharmacists, 545/873, 62.4%, were accepted by prescribers, 14/873, 1.6% were not accepted by the prescribers, while 228/873, 26.1% had unknown outcomes at the time of data entry (Table 3).

The majority of interventions documented were from the Neonatal Intensive Care Unit (SCN 3 with CSF level 6) (343/873, 39.1%), involving medications such as gentamicin (46/343, 13.4%), benzylpenicillin (37/343, 10.8%), caffeine (34/343, 9.9%), parenteral nutrition (23/343, 6.7%), and morphine (16/343, 4.7%) and meropenem (16/343, 4.7%), with 343 as the filtered subset of intervention records with complete medication in the Neonatal Intensive Care Unit (Table 4). Medications documented unique to the Neonatal Intensive Care Unit (SCN 3) included parenteral nutrition, dexamethasone, alprostadil, dobutamine, magnesium sulfate, and paracetamol. The interventions involving these medications were also rated as high- or extreme risk by the pharmacists.

**Figure 1:**
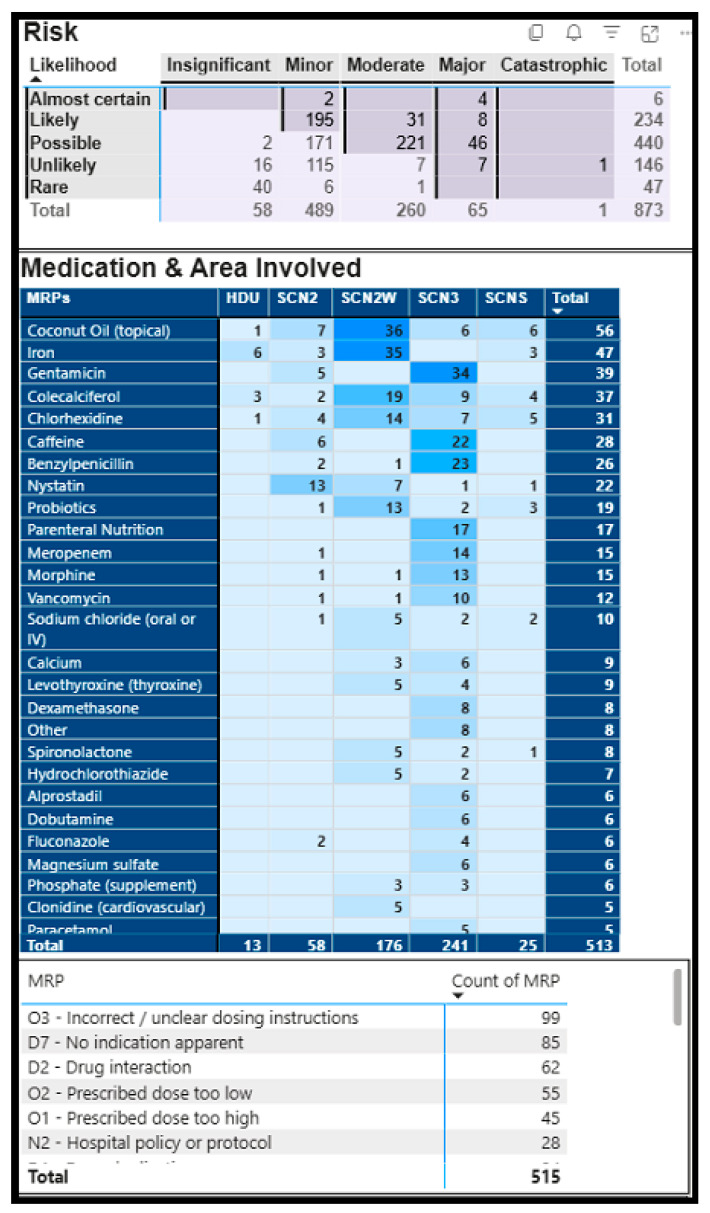
Medication Related Problems rated as High and Extreme Risk using Risk Assessment Matrix, assessing the likelihood and consequence/impact of an MRP if the pharmacist’s intervention is not made. Note: Figures with darker legends were interventions rated as high and extreme risk according to risk matrix [21,27].

A total of 515/873, 59.0% MRPs were rated as high- and extreme-risk (Figure 1). Medications involved in high- and extreme-risk included intravenous antibiotics (gentamicin, benzylpenicillin, meropenem, vancomycin), coconut oil, iron, caffeine, parenteral nutrition, and morphine (Figure 1).

## 4. Discussion

This study evaluated pharmacist intervention patterns in a neonatal unit. Compared with a previous study conducted at the same hospital, the pharmacist intervention rate increased from 1.0 intervention per 100 medication charts reviewed to 3.1 interventions per 100 medication charts [21]. The continuous improvement in the pharmacy clinical interventions documentation and analysis is considered to be one of the contributing factors to the increased intervention rates in this study compared to the previous study, which involved paper documentation and transcription onto an Excel spreadsheet [21,27].

MRPs involving medication dose remained the most common MRPs, although reduced from our previous study of 47.7% of interventions documented in January 2005 to December 2014 to 36.7% of the pharmacist interventions [21]. Other studies on medication errors showed similar trends of medication dose-related errors being the most common MRP, with 61.9% dose-related interventions in a study in Egypt, 46.1% in a Korean study, and other previously reported literature [6,11,28]. Pharmacist interventions capturing MRPs related to hospital policy or protocols have increased to 17.4% from 10.7%, while the pharmacist interventions involving laboratory monitoring have been relatively consistent in this study, 4.6% compared to 4.9% [21].

Following pharmacist interventions, 454/873, 52.0% of dose-related MRPs were referred to the prescriber. Of these, 85/873, 9.7% of medication doses were increased, while 65/873, 7.4% of medication doses were decreased. In addition to medication review, pharmacists also provided education or information on medications in 85/873,9.7% of cases. The overall acceptance rate of pharmacist clinical interventions was 62.4%, which was lower than reported in a comparable 4-month intervention study with a 98.8% acceptance rate and a 6-month study reporting a 95.2% prescriber acceptance rate [6,11]. Outcomes were recorded as unknown for 26.1% of interventions at the time of documentation. This may have contributed to the lower observed acceptance rate, as interventions were documented daily at the study site, while follow-up outcomes were often recorded in patient clinical notes rather than in the intervention database.

Similarly to previous studies carried out in the unit, the high- and extreme-risk MRPs on neonatal wards involved high-risk medications including antimicrobials, e.g., caspofungin, vancomycin, and gentamicin, morphine, dobutamine, and total parenteral nutrition [21,27]. This is consistent with previous reports discussing the complexity of medication use in neonates with the increased utilization of high-risk medications [29,30].

The pharmacy intervention trend analysis highlighted an important change in clinical practice associated with a specific medication. Coconut oil, used to maintain and improve skin integrity in preterm infants less than 30 weeks of gestation, has a complex dosing regimen based on neonatal gestational age (25 weeks of gestation or less or greater than 25 weeks of gestation) and whether the infant is in or out of an incubator, which accounted for the highest MRPs. The introduction of multiple dosing variables increases the risk of prescribing and administration errors, particularly in a high-acuity neonatal intensive care setting. Coconut oil was not reported in the earlier study, as it had not yet been introduced into routine clinical practice [21,27]. While the clinical consequence or impact for MRPs on coconut oil is low, the high incidence of pharmacist interventions (101/873, 11.6%) has rendered it one of the high-risk MRPs in the neonatal unit according to the risk matrix. In contrast, iron therapy remained a consistent contributor across both studies, with treatment protocols largely unchanged, and continued to represent the second most common medication associated with MRPs [21,27].

Although the neonatal pharmacist at the study site does not have the capacity to participate in the daily ward round, the pharmacist participates in the weekly multidisciplinary grand rounds and the monthly Neonatal Coordinating Group meeting. Ward rounds would enable the pharmacist to rapidly identify medication errors during the prescribing phase and provide real-time recommendations to prescribers [7,10]. Pharmacist participation in the grand rounds, directorate meeting, as well as the delivery of medication safety education to the multidisciplinary team at the study site, has created a platform for the pharmacist to provide feedback, facilitate discussion of medication incident trends and strategies, and improve medication safety.

Pharmacists further contribute to improving medication safety by coordinating the review, development, and publication of site-specific neonatal medication protocols [25]. In developing these protocols, established neonatal medicine references, such as Neofax^®^ and the British National Formulary for Children, and medication monographs from other institutions, such as the Australasian Neonatal Medicines Formulary (ANMF) [31], were critically reviewed and considered when compiling the site-specific medication monographs. This process is undertaken with multidisciplinary input, including medical review and formal endorsement at the monthly directorate meeting. The resulting site-specific medication monographs provide standardized guidance on dosing, administration, and monitoring, supporting safe and consistent medication use across the neonatal unit. [18,19,20]. Limitations of the study include the underreporting of interventions by the pharmacists and the variability between individual pharmacists in the documentation of clinical interventions. Measures in place to address such limitations include the continuous monitoring and analysis of pharmacist interventions in the study site. This long -term data provides a greater number of records to enable in-depth statistical analyses and examination of trends.

## 5. Conclusions

Our study demonstrates that hospital pharmacists play an integral role in ensuring neonatal medication safety and contribute to the reduction in MRPs within a neonatal unit. Regular analysis of pharmacist interventions provides valuable insights into local MRP trends and highlights opportunities for quality improvement and education.

## Figures and Tables

**Table 1:** Medication-Related Problem documented by the pharmacist in the Neonatal Clinical Care Unit.

MRP	Number of MRP	% of MRP
Hospital policy or protocol	152	17.4%
Incorrect/unclear dosing instructions	134	15.3%
No indication apparent	106	12.1%
Prescribed dose too low	92	10.5%
Drug interaction	66	7.6%
Prescribed dose too high	66	7.6%
Laboratory monitoring	40	4.6%
Preventative therapy required	32	3.7%
Drug duplication	29	3.3%
Other dose issue	28	3.2%
Condition untreated	24	2.7%
Other drug selection issue	21	2.4%
Other education or information service provided	20	2.3%
Contraindications apparent	16	1.8%
Wrong drug	10	1.1%
Inappropriate dosage form	7	0.8%
Overuse by patient	5	0.6%
Incorrect strength	5	0.6%
Condition undertreated	5	0.6%
Other untreated indication and/or issue	3	0.3%
Prescribing omission of regular medications	3	0.3%
Technician intervention	2	0.2%
Other toxicity/Adverse Drug Reaction (ADR) issue	2	0.2%
Other compliance issue	1	0.1%
Patient requests medication information	1	0.1%
Other monitoring required/recommended	1	0.1%
Not classifiable under another category	1	0.1%
Toxicity, allergic reaction, or ADR present	1	0.1%
Total	873	

**Table 2:** Pharmacist Recommendation (PR) following identifying Medication Related Problems in the Neonatal Clinical Care Unit.

Pharmacist Recommendation (PR)	Count of PR	% of PR
Refer to prescriber	454	52.0%
Dose increase	85	9.7%
Dose decrease	65	7.4%
Provided other information	58	6.6%
Dose schedule/frequency change	39	4.5%
Prescription not dispensed	39	4.5%
Refer to hospital	36	4.1%
Other changes to therapy	29	3.3%
Provide written summary of medication/s	25	2.9%
Laboratory monitoring	11	1.3%
Other referral required	9	1.0%
Formulation change	8	0.9%
Drug change	6	0.7%
Non-laboratory monitoring	4	0.5%
Refer to pharmacist (when identified by intern/technician)	2	0.2%
Provide education or counseling session	2	0.2%
Refer to another pharmacist	1	0.1%
Total	873	

**Table 3:** Action Taken Following Pharmacist Recommendation.

Action	Count of Action	% of Action
Prescriber has accepted recommendation	545	62.4%
Unknown at time of data entry	228	26.1%
Pharmacist has provided service as recommended	77	8.8%
Prescriber has NOT accepted recommendation	14	1.6%
Pharmacist has accepted recommendation	6	0.7%
Patient has accepted pharmacist recommendation	3	0.3%
Total	873	

**Table 4:** Medications involved in the Neonatal Intensive Care Unit pharmacist interventions.

Ward/Area Value	SCN3 *	SCN2 *	HDU *	SCN2W *	SCNS *	Total
MRPs						
Coconut Oil (topical)	15	20	2	58	6	101
Colecalciferol	17	23	5	59	7	111
Iron	3	15	9	65	4	96
Gentamicin	46	8	1			55
Probiotics	8	15	1	23	7	54
Chlorhexidine	11	16	1	18	6	52
Nystatin	9	30		12	1	52
Caffeine	34	14				48
Benzylpenicillin	37	7		3		47
Parenteral Nutrition	23	2				25
Morphine	16	1		1		18
Meropenem	16	1				17
Other	9			4		13
Vancomycin	10	1		1		12
Calcium	6			5		11
Levothyroxine (thyroxine)	5			5		10
Paracetamol	6			4		10
Sodium chloride (oral or IV)	2	1		5	2	10
Chloramphenicol	5	2		2		9
Dexamethasone	9					9
Hydrochlorothiazide	2	1		5		8
Spironolactone	2			5	1	8
Fluconazole	5	2				7
Alprostadil	6					6
Dobutamine	6					6
Magnesium sulfate	6					6
Phosphate (supplement)	3			3		6
Clonidine (cardiovascular)				5		5
Benzathine benzylpenicillin	2	2				4
Caspofungin	4					4
Heparin	4					4
Omeprazole				4		4
Other drugs for electrolyte imbalance	1	1	2			4
Oxycodone				4		4
Pregabalin				4		4
Tapentadol				4		4
Nystatin (skin)		2		1		3
Aciclovir	2					2
Epoetin alfa	2					2
Flucloxacillin		2				2
Probiotic				2		2
Sodium chloride	2					2
Azithromycin	1					1
Bosentan				1		1
Cefazolin	1					1
Cefotaxime				1		1
Diphtheria, tetanus, and pertussis vaccines	1					1
Fentanyl				1		1
Folic acid				1		1
Furosemide (frusemide)	1					1
Levetiracetam	1					1
Linezolid	1					1
Meningococcal vaccines		1				1
Mupirocin (skin)	1					1
Pentoxifylline (oxpentifylline)	1					1
Phenobarbital (phenobarbitone)	1					1
Tamsulosin				1		1
Tramadol				1		1
Total	343	167	21	308	34	873

* SCN 3 Level 3 neonatal unit (Neonatal Intensive Care Unit) (CSF level 6); other neonatal units are Level 2 neonatal unit (CSF level 4 and 5) [23,24].

## Data Availability

The original contributions presented in this study are included in the article. Further inquiries can be directed to the corresponding author.

## Abbreviations

The following abbreviations are used in this manuscript:

ADR	Adverse Drug Reaction
CSF	Clinical Significance Framework
HDU	High Dependency Unit
MRP	Medication Related Problems
NICU	Neonatal Intensive Care Unit
PR	Pharmacist Recommendation
REDCap	Research Electronic Data Capture
SCN	Special Care Nursery
TPN	Total Parenteral Nutrition

## References

[1] Roughead E.E., Semple S.J., Rosenfeld E. (2016). The extent of medication errors and adverse drug reactions throughout the patient journey in acute care in Australia. JBI Evid. Implement..

[2] Dooley M., Bennett G., Clayson-Fisher T., Hill C., Lam N., Marotti S., O’Hara K., Potts C., Shum B., Tong E. (2024). Advanced pharmacy Australia clinical pharmacy standards. J. Pharm. Pract. Res..

[3] Krzyzaniak N., Bajorek B. (2017). A global perspective of the roles of the pharmacist in the NICU. Int. J. Pharm. Pract..

[4] Yalçın N., Kaşıkcı M., Çelik H.T., Allegaert K., Demirkan K., Yiğit Ş. (2023). Impact of clinical pharmacist-led intervention for drug-related problems in neonatal intensive care unit a randomized controlled trial. Front. Pharmacol..

[5] Dwivedi P., Sah S.K., Murthy S., Ramesh M. (2024). Identification and resolution of drug-related problems among neonates in neonatal intensive care unit (NICU): A prospective longitudinal observational study. Clin. Epidemiol. Glob. Health.

[6] Ahmed N.A., Fouad E.A., El-Asheer O.M., Ghanem A.S.M. (2024). Pharmaceutical interventions for drug-related problems in the neonatal intensive care unit: Incidence, types, and acceptability. Front. Pharmacol..

[7] De Jager Z., Schellack N., Gous A. (2014). What role does the clinical pharmacist play in the neonatal intensive care unit?. SA Pharm. J..

[8] Sanghera N., Chan P.-Y., Khaki Z.F., Planner C., Lee K.K.C., Cranswick N., Wong I.C.K. (2006). Interventions of hospital pharmacists in improving drug therapy in children: A systematic literature review. Drug Saf..

[9] Naseralallah L.M., Hussain T.A., Jaam M., Pawluk S.A. (2020). Impact of pharmacist interventions on medication errors in hospitalized pediatric patients: A systematic review and meta-analysis. Int. J. Clin. Pharm..

[10] Drovandi A., Robertson K., Tucker M., Robinson N., Perks S., Kairuz T. (2018). A systematic review of clinical pharmacist interventions in paediatric hospital patients. Eur. J. Pediatr..

[11] Chedoe I., Molendijk H.A., Dittrich S.T., Jansman F.G., Harting J.W., Brouwers J.R., Taxis K. (2007). Incidence and nature of medication errors in neonatal intensive care with strategies to improve safety: A review of the current literature. Drug Saf..

[12] Chedoe I., Molendijk H., Hospes W., Van den Heuvel E.R., Taxis K. (2012). The effect of a multifaceted educational intervention on medication preparation and administration errors in neonatal intensive care. Arch. Dis. Child. Fetal Neonatal Ed..

[13] Nguyen M.N.R., Mosel C., Grzeskowiak L.E. (2018). Interventions to reduce medication errors in neonatal care: A systematic review. Ther. Adv. Drug Saf..

[14] Campino A., Lopez-Herrera M.C., Lopez-de-Heredia I., Valls-i-Soler A. (2009). Educational strategy to reduce medication errors in a neonatal intensive care unit. Acta Paediatr..

[15] Jaam M., Naseralallah L.M., Hussain T.A., Pawluk S.A. (2021). Pharmacist-led educational interventions provided to healthcare providers to reduce medication errors: A systematic review and meta-analysis. PLoS ONE.

[16] Nundeekasen S., McIntosh J., McCleary L., O’Neill C., Chaudhari T., Abdel-Latif M.E. (2024). Voluntary Neonatal Medication Incident Reporting—A Single Centre Retrospective Analysis. Healthcare.

[17] Krzyzaniak N., Bajorek B. (2016). Medication safety in neonatal care: A review of medication errors among neonates. Ther. Adv. Drug Saf..

[18] Kane-Gill S.L.P., Dasta J.F.M., Buckley M.S.P., Devabhakthuni S.P., Liu M.P., Cohen H.P., George E.L.C.-K., Pohlman A.S.M., Agarwal S., Henneman E.A.P. (2017). Clinical practice guideline: Safe medication use in the ICU. Crit. Care Med..

[19] Flint R.B., van Beek F., Andriessen P., Zimmermann L.J., Liem K.D., Reiss I.K., de Groot R., Tibboel D., Burger D.M., Simons S.H. (2018). Large differences in neonatal drug use between NICUs are common practice: Time for consensus?. Br. J. Clin. Pharmacol..

[20] Lucas A.J. (2004). Improving medication safety in a neonatal intensive care unit. Am. J. Health-Syst. Pharm..

[21] Teoh S.W., Hattingh L., Lebedevs T., Parsons R. (2017). Analysis of clinical intervention records by pharmacists in an Australian principal referral and specialist women’s and newborns’ hospital. J. Pharm. Pract. Res..

[22] Levine S.R., Cohen M.R., Blanchard N.R., Federico F., Magelli M., Lomax C., Greiner G., Poole R.L., Lee C.K.K., Lesko A. (2001). Guidelines for preventing medication errors in pediatrics. J. Pediatr. Pharmacol. Ther..

[23] Tiny Sparks W.A. Maternity & Neonate Hospitals in Western Australia. https://www.tinysparkswa.org.au/maternity-hospitals-in-wa.

[24] Child and Adolescent Health Service Neonatal Units. Government of Western Australia. https://cahs.health.wa.gov.au/Our-services/Neonatology/Neonatal-Units.

[25] King Edward Memorial Hospital Neonatal Medication Protocols. North Metropolitan Health Service: Government of Western Australia. https://wnhs.health.wa.gov.au/For-Health-Professionals/Clinical-Guidelines/Neonatal.

[26] Sajogo M., Teoh S.W.K., Lebedevs T. (2023). Pharmacist clinical interventions: Five years’ experience of an efficient, low-cost, and future-proofed tool. Res. Soc. Adm. Pharm..

[27] Frestel J., Teoh S.W.K., Broderick C., Dao A., Sajogo M. (2023). A health integrated platform for pharmacy clinical intervention data management and intelligent visual analytics and reporting. Explor. Res. Clin. Soc. Pharm..

[28] Kim Y., Rho J., Suh Y., Choi K., Lee E., Lee E., Choi C.W. (2018). Pharmacist Interventions in Neonatal Intensive Care Unit and Associated Cost Avoidance and Cost Savings. J. Korean Soc. Health-Syst. Pharm..

[29] Fernandez-Llamazares C.M., Calleja-Hernández M., Manrique-Rodríguez S., Pérez-Sanz C., Durán-García E., Sanjurjo-Sáez M. (2012). Prescribing errors intercepted by clinical pharmacists in paediatrics and obstetrics in a tertiary hospital in Spain. Eur. J. Clin. Pharmacol..

[30] Krzyżaniak N., Pawłowska I., Bajorek B. (2018). The role of the clinical pharmacist in the NICU: A cross-sectional survey of Australian and Polish pharmacy practice. Eur. J. Hosp. Pharm..

[31] Shaniv D., Bolisetty S., Young T.E., Mangum B., Ainsworth S., Elbers L., Allegaert K. (2023). neonatal drug formularies—A global scope. Children.

